# Supplementary material for: Content-rich biological network constructed by mining PubMed abstracts
Source: BMC Bioinformatics. 2004 Oct 8;5:147. doi: 10.1186/1471-2105-5-147 (PMC528731; doi:10.1186/1471-2105-5-147)
Supplement: Additional File 2 — The original results of the above study (non-essential files are deleted to keep the file size under the limit set by BMC bioinformatics). [file 1471-2105-5-147-S2.bz2 › chilibotAdditionalFile2/dip05/626ID10924114E11013/html/MUTB_MUTA.html]

 


 **MUTB** and **MUTA** 
  
Found 3 abstracts in PubMed, retrieved 3.  
 

 What does Google say? 
 PDF only 
| .edu only 

---

**Interactive relationship** (e.g. stimulation, inhibition, etc)

**Non-interactive relationship** (e.g. studied together, co-existance, homology, etc.)

- The  **mutA**  and  **mutB**  gene products were detected in Western blots immunoblots with mutase specific antibodies and by direct detection of mutase activity with a newly developed assay method.  Ref: 8099072 J Bacteriol, 1993
- The two largest  **mutA**  and  **mutB**  overlap by 1 nucleotide and encode proteins of 616 and 733 residues showing high amino acid sequence similarities to each other and to methylmalonyl CoA mutases from P. shermanii and mammalian sources.  Ref: 8099072 J Bacteriol, 1993
- The transcriptional start of the  **mutA**   **mutB**  message, determined by S1 mapping, coincides with the first nucleotide of the translational start codon.  Ref: 8099072 J Bacteriol, 1993
- To investigate the structure function relationships of CR 20beta HSDs, we generated mutants by site directed mutagenesis at the following positions  **MutA**  I15T,  **MutB**  T15I, and  **MutB**  Q27K.  Ref: 10725344 Proc Natl Acad Sci U S A, 2000
